# The epidemiology of acromioclavicular joint excision

**DOI:** 10.1177/2309499018816521

**Published:** 2018-12-09

**Authors:** Michael McLean, Katie Hoban, Rohit Gupta, Anthony Gibson, Andrew J. Brooksbank, Umberto G. Fazzi, Angus Arthur, David Martin, Paul J. Jenkins, Neal L. Millar

**Affiliations:** 1 Institute of Infection, Immunity and Inflammation, College of Medicine, Veterinary and Life Sciences, University of Glasgow, Glasgow, UK; 2 Department of Orthopaedic Surgery, Queen Elizabeth University Hospital, Glasgow, UK; 3 Department of Orthopaedic Surgery, Glasgow Royal Infirmary, Glasgow, UK

**Keywords:** acromioclavicular arthritis, acromioclavicular joint excision, epidemiology, service planning, shoulder, urban population

## Abstract

**Background:**

With the development of arthroscopic procedures such as subacromial decompression (ASAD) and rotator cuff repair (RCR), it is hypothesized that there may have been a similar rise in the performance of acromioclavicular joint excision (ACJE). The purpose of this study was to investigate the epidemiology of ACJE to examine incidence, surgical technique, age, gender of patients and associated procedures in an urban population.

**Methods:**

A prospectively collected surgical database was retrospectively examined to identify patients undergoing ACJE. Associated procedures such as ASAD or RCR were determined from these records. The demographic details (age and gender) were also recorded.

**Results:**

A total of 411 ACJEs were performed over the study period (n = 216 males, n = 195 female). The overall incidence increased from 9.3 per 100,000 in 2009, to a peak of 19.6 per 1,00,000 in 2013. In 349 patients, ACJE was undertaken as part of an arthroscopic procedure, of which 332 were ASAD+ACJE alone. The prevalence of arthroscopic ACJE in ASADs was 23.7% (349/1400). ACJE was performed as an open procedure in 62 (15%) cases. Those undergoing open ACJE were younger than those undergoing an arthroscopic procedure (mean difference 6.2 years, 95% CI 3.2–9.2, p < 0.001).

**Conclusions:**

We demonstrate an increasing incidence of ACJE in the general population. The groups of patients most likely to undergo ACJE are women aged between 45 and 54 years old, men aged 55–64 years and the most socioeconomically deprived. The higher incidence of ACJE in the most deprived socioeconomic quintile may have public health implications. Level of Evidence: II; retrospective design: prognosis study.

## Introduction

The acromioclavicular joint (ACJ) is a diarthroidal, encapsulated, hyaline cartilage lined and meniscal complex interposed articulation. This relationship completes a clavicular strut between thorax and shoulder girdle. The coracoclavicular ligament complex (trapezoid and conoid ligaments) permits synchronous scapuloclavicular motion, with minimal ACJ motion (5–8°).^1^ The ACJ is vulnerable to both traumatic injury and degenerative disease. ACJ disruption is commonly seen in clinical practice, accounting for 12% of injuries to the shoulder girdle.^2^,^3^ This likely underestimates the true incidence; however, the majority (2:1) of these injuries are incomplete separations (sprains and subluxations).^4^

A suggested aetiology of primary ACJ osteoarthritis (OA) is the transmission of a high axial load through the small joint surface area (average 9 × 19 mm^2^), leading to early failure (OA or osteolysis).^5,6^ Risk factors associated with developing secondary ACJ OA include occupational heavy lifting, manual work, repetitive micro-trauma (weight lifting, swimming, basketball), inflammatory arthropathies, septic arthritis, instability and traumatic injury.^7^

ACJ OA typically presents in the 5th decade and usually without any history of traumatic injury. The pain is located over the ACJ itself, exacerbated by cross-body abduction, behind back motion and overhead reaching.^8^ More specific signs include a painful arc, cross arm abduction and joint line tenderness.^9^ Combined clinical examination and radiographic review of 310 shoulder joints, in patients over 50, reported an incidence of painful ACJ OA in 45% of males and 42% of female with radiographic incidence of 57% and 54%, respectively.^10^ MRI scanning of asymptomatic patients has shown features of ACJ OA in 48% (<30 years) and 82% (>30 years).^11^ It appears while the prevalence of asymptomatic ACJ OA can be described radiologically, this may not be clinically relevant without clinical correlation.

The management ACJ OA includes non-operative treatments such as rest, analgesia, anti-inflammatory medication and local anaesthetic with corticosteroid injection. Operative treatments are typically utilized after 6 months of failed non-operative treatment and include open or arthroscopic distal clavicle excision.^12^ With the development of arthroscopic procedures such as subacromial decompression (ASAD) and rotator cuff repair (RCR), it is hypothesized that there may have been a similar rise in the performance of ACJ excision (ACJE).^13^ Quantifying the rate of ACJE is important in the understanding the natural history of ACJ OA defining surgery as the final end point. The aim of this study was to investigate the epidemiology of ACJE to examine incidence, surgical technique, age, gender of patients and associated procedures in an urban setting.

## Material and methods

Research ethics committee (REC) approval was not required as there was no contact with patients, allocation or concealment of treatment and only routine outcome metrics were collected such as demographics and incidence.

A retrospective analysis study was performed over a 6-year period (2009–2014), in two adjacent UK-based metropolitan university teaching hospitals. These units provided primary, secondary and tertiary orthopaedic services. Research ethics committee approval was not required as there was no contact with patients, allocation or concealment of treatment and only routine outcome metrics were collected such as demographics and incidence.

Electronic patient records were used to identify patients undergoing ACJE between 2009 and 2014 on our prospectively recorded database and electronic record system (Bluespier, Worcestershire, UK). The nature of the procedure (open or arthroscopic) and associated procedures such as ASAD and/or RCR were determined from these records. The demographic details (age and gender) were also recorded.

Population incidences were calculated using the mid-year population estimates for the combined catchment area of both hospitals. The total adult (15+ years) population was 475,147. These data were supplied from the Health Board Business Intelligence Department.^14^ These were divided into 5 and 10 year age ranges. The incidence was defined as the number of patients undergoing ACJE surgery in a year, divided by the annual eligible population. Ninety-five per cent confidence intervals were calculated using the following formula: Ö(*p*(1 − *p*)/*n*), where *p* = incidence (as a decimal proportion) and *n* = population size. Patients may attend our institutions from outwith the catchment area. This population was also estimated in the population data from Business Intelligence and defined as ‘cross-boundary population’. The proportion of patients in our data set from outwith the catchment area was calculated and compared with the population estimates.

The data set was analysed using the statistical package SPSS version 19 (v19, SPSS Inc, Illinois). Descriptive statistics (mean, range and standard deviation, SD) were calculated. The data were assessed for normality using histograms, and parametric tests were used. The annual incidence was calculated as simple proportions. The trend in incidence over time was calculated using the Spearman correlation coefficient.

## Results

**Figure 1.**
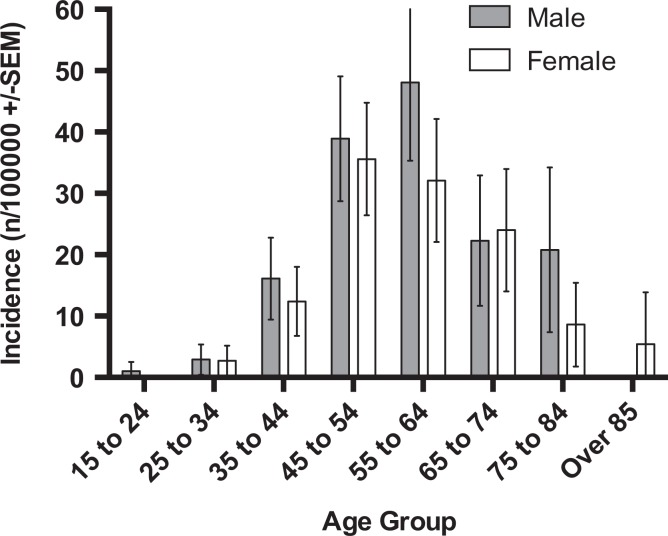
Incidence of ACJE by age and gender. ACJE: acromioclavicular joint excision.

**Figure 2.**
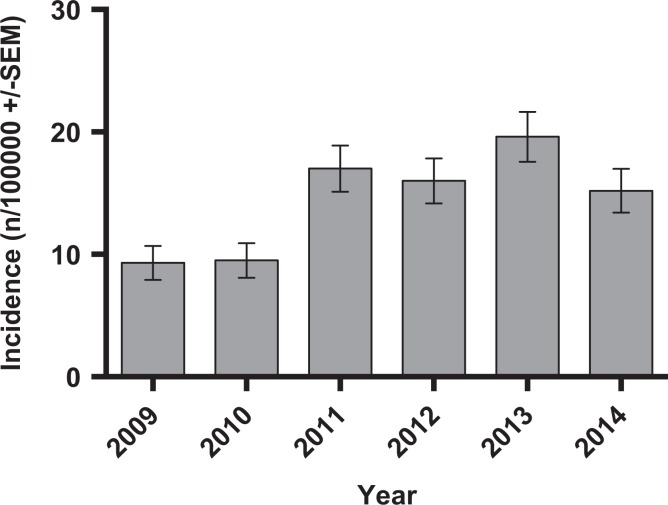
Incidence of ACJE by year. ACJE: acromioclavicular joint excision.

**Figure 3.**
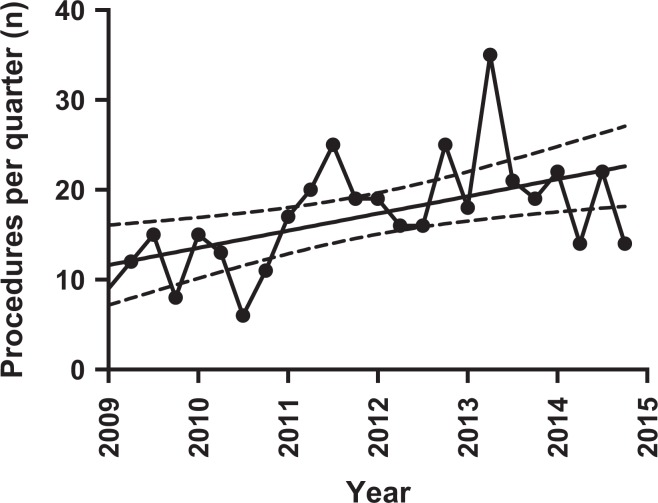
Change in number of ACJE procedure performed over time (Spearman *r* = 0.573, *p* = 0.003, solid line = linear regression). ACJE: acromioclavicular joint excision.

A total of 411 ACJEs were performed over the study period (*n* = 216 males, *n* = 195 female). The overall male incidence was 19.1 per 100,000 and 15.7 per 100,000 in females (OR 1.19, 0.98–1.45, *p* = 0.075) (Table 1; Figure 1). The overall incidence increased from 9.3 per 100,000 in 2009 to a peak of 19.6 per 100,000 in 2013 (Figure 2). The incidence increased by an average of 1.9/100,000/year over the study period (Spearman *r* = 0.573, *r* ^2^ = 0.288, *p* = 0.003, Figure 3). One hundred forty-six (35.5%) patients were from the most deprived socioeconomic quintile (Table 2). Eighty-five per cent of patients were in the American Society of Anesthetists (ASA) 1 and 2 categories (Table 2). In 349 patients ACJE was undertaken as part of an arthroscopic procedure, of which 332 were ASAD+ACJE alone (ACJ arthritis diagnosed clinically and radiologically prior to surgery). The prevalence of arthroscopic ACJE in ASADs was 23.7% (349/1400). An additional capsular release was performed in two (0.6%) patients. The prevalence of arthroscopic ACJE was lower in patients undergoing arthroscopic RCR (*n* = 8, prevalence = 1.9%) compared with a cuff debridement in nine cases (*n* = 9, prevalence = 23.7%) (OR 0.06, 95% CI 0.02–0.17, *p* < 0.001). Patients with a rotator cuff tear were old than those without by a mean 8.3 years (95% CI 4.7–12.0, *p* < 0.001, Table 3). ACJE was performed as an open procedure in 62 (15%) cases. The rate of open surgery did not change over the time period (*p* = 0.816). Those undergoing open ACJE were younger than those undergoing an arthroscopic procedure (mean difference 6.2 years, 95% CI 3.2–9.2, *p* < 0.001).

**Table 1.** Incidence of ACJE in the population served by the two study institutions.

Age group	Male			Female		
	ACJE	Population	Incidence (*n*/100,000)	ACJE	Population	Incidence (*n*/100,000)
15–24	2	41299	1.0	0	41,282	0
25–34	7	47720	2.9	6	44,347	2.7
35–44	29	36081	16.1	24	38,832	12.4
44–54	73	37577	28.9	75	42,148	35.6
55–64	71	29527	48.1	51	31,796	32.1
65–74	22	19708	22.3	29	24,172	24
75–84	12	11536	20.8	8	18,544	8,6
Over 85	0	3103	0	2	7475	5.4
Overall	216	226551	19.1	195	24,8596	15.7

ACJE: acromioclavicular joint excision.

**Table 2.** Gender distribution of procedure type, socioeconomic deprivation quintile (SIMD) and comorbidity (ASA).^a^

	Male (*n* = 216)	Female (*n* = 195)	*p* Value
Procedure			
Open (*n* = 62)	37 (17.1%)	25 (12.8%)	*p* = 0.223
ASAD+ACJE (*n* = 349)	179 (82.9%)	170 (87.2%)	
SIMD			
1 (*n* = 146)	76 (52.1%)	70 (47.9%)	*p* = 0.921
2 (*n* = 77)	44 (57.1%)	33 (42.9%)	
3 (*n* = 74)	37 (50%)	37 (50%)	
4 (*n* = 27)	27 (50.9%)	26 (49.1%)	
5 (*n* = 58)	30 (51.7%)	28 (48.3%)	
Unknown (*n* = 3)			
ASA			
1	84 (56.4%)	65 (43.6%)	*p* = 0.149
2	97 (48.5%)	103 (51.5%)	
3	23 (65.7%)	12 (34.3%)	
4	0	0	
Unknown (*n* = 27)			

ASA: American Society of Anesthetists; SIMD: Scottish Index of Multiple Deprivation.

^a^ *p* values indicate *χ* ^2^ test.

**Table 3.** Age compared across gender, procedure type and if the rotator cuff was repaired/debrided.

	Age (years, mean (SD))	*p* Value
Gender		
Male	54 (11.5)	*p* = 0.463
Female	54.8 (11.0)	
Procedure type		
ASAD+ACJE	55.3 (11.0)	*p* < 0.001
Open	49.1 (11.4)	
Cuff status		
Cuff tear absent	53.6 (11.0)	*p* < 0.001
Cuff tear present	62.0 (11.4)	

ACJE: acromioclavicular joint excision; ASAD: arthroscopic subacromial decompression.

**Figure 4.**
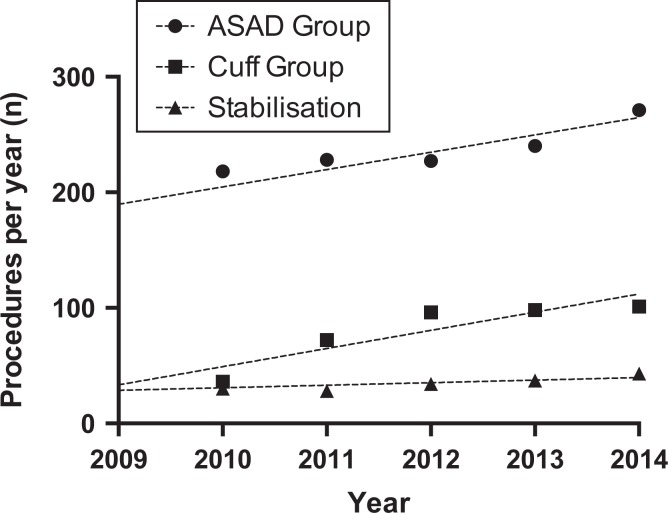
Number of other index shoulder arthroscopic procedures performed per year (lines represent linear regression).

**Figure 5.**
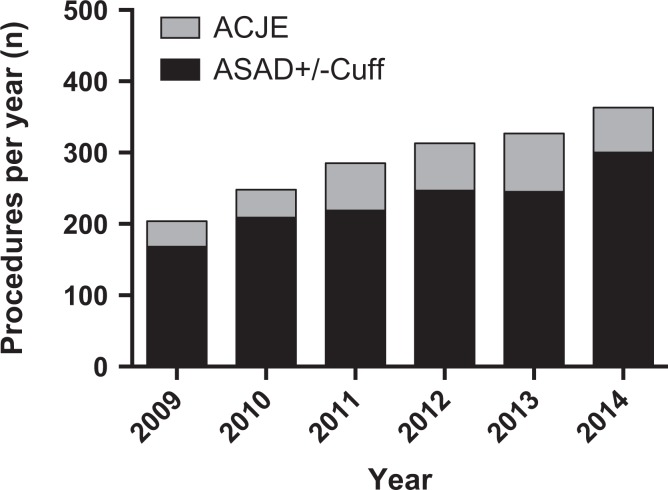
Proportion of arthroscopic ACJE compared with ASAD (+/− associated cuff procedure) during study period. ACJE: acromioclavicular joint excision; ASAD: arthroscopic subacromial decompression.

During the study period, there were 1051 isolated ASADs performed (Figure 4). The number increased from 159 per year in 2009 to 196 in 2014 (Spearman *r* = 0.943, *p* = 0.017, Figure 4). A similar trend was seen in rotator cuff procedures, which increased from 33 to 101 (Spearman *r* = 1.00, *p* = 0.003, Figure 4). The total number of arthroscopic RCRs was 426, while the cuff was debrided in 38 cases. The number of arthroscopic stabilization procedures remained static (*n* = 205, Spearman *r* = 0.771, *p* = 0.103, Figure 4). The proportion of ACJEs performed compared with ASADs remained static (*χ* ^2^ test for trend, *p* = 0.464; Figure 5).

The mean age of males was 54 years (range 35–84), with peak incidence in the 55–64 age bracket (48.1 per 100,000, Table 3). The mean age of females was 54.8 years (range 35–84) with the peak incidence in the 44–54 age group (35.6 per 100,000, Tables 2 and 3). There was no significant difference in the mean age between groups (mean difference −0.8, 95% CI −3.0 to 1.4, *p* = 0.463).

## Discussion

To our knowledge, this is the first study to systematically explore the epidemiology of ACJE. We demonstrate an increasing incidence of ACJE in the general population. The groups of patients most likely to undergo ACJE are women aged between 45 and 54 years old, men aged 55–64 years and the most socioeconomically deprived. There was no statistical difference in the incidence with respect to sex and mean age. Unlike arthroscopic ACJE, the rate of open ACJE was been stable over the same time period. This highlighted a concomitant rise in arthroscopic surgery in general, particularly ASAD and ARCR. Those undergoing open surgery had no associated rotator cuff tears and were younger. Additionally, ACJE as a proportion of ASAD+/− rotator cuff treatment remained stable. RCR, biceps tenotomy and biceps tenodesis were undertaken as associated procedures in a small proportion of cases.

Age is important in understanding the epidemiology of ACJ excision. We know that symptoms and radiological features of ACJ arthritis are more common over the age of 30 and peaks at the 5th decade.^8,10^ The mean age for males and females undergoing ACJE is 54 years old and the peak age of onset between 45 and 64. This age range accounted for 77% of the total number of ACJE’s on men and 65% on women. Given the rising incidence of 1.9/100,000/year during the study period, we would ask if there was any change in population demographics during this time frame. The total population used – 475,147 – was a static figure. However, recent government data have shown a 3.5% growth in NHS Greater Glasgow & Clyde health board between 2005 and 2015.^20^ In addition, the 45–75 age group now comprises >37.5% of the total population demographic, a rise of 3%. As a result of the baby-boomer generation, there has been a rise of 11% in the 45–59 age group and 18% in the 60–75 age group. A baby born on January 1st 1960 would be 54 by the end of this study period. This may account for part of rise in ACJE incidence over this frame. Interestingly, this effect would be expected to slightly rise over the next 5 years before falling.^14^

The additional ACJE increase in incidence not attributable to the ageing population may be explained by the overall rise in arthroscopic shoulder procedures. This has been observed elsewhere and these findings echoed in our study.^13^ No significant increase in ACJE+/−ASAD as a proportion of total arthroscopic ASAD may be explained by the rise of arthroscopic surgery. Possible reasons include, but not limited to; increase in the number of specialist shoulder surgeons, surgical preference, increased surgical training and exposure to arthroscopic surgery, benefits of arthroscopy for example, enhanced rehabilitation and patient preference.^7^

The higher incidence of ACJE in the most deprived socioeconomic quintile may have public health. A proposed pathological mechanism leading to ACJ OA is high force transmission through a small surface area. You could postulate that either injury or lifetime high load transmission would increase the risk of pathology. There are established risk factors (non-mechanical and mechanical) for the development of both regional shoulder pain and symptomatic ACJ OA. Non-mechanical risk factors include poor diet, reduced leisure time physical exercise, obesity, smoking, alcohol consumption, stress and high psychosocial job demand.^15,16^ Similarly, multiple studies show clear associations between mechanical risk factors as demonstrated by Linekar et al.^17^ This also highlighted a host of patient factors – repetitive lifting, pushing, pulling and working above shoulder height. Previous studies have demonstrated this relationship with diagnoses of subacromial impingement syndrome and rotator cuff tendinopathy.^18^ We also know that manual work and total lifetime weight lifted are significant risk factors for developing symptomatic ACJ OA.^7^ However, our study did not attempt to delineate the reason why there is a higher incidence of ACJ OA in social deprivation quintiles. Evidence suggests risk factors (as discussed above) are more prevalent in lower social deprivation quintiles^19^ and we acknowledge that further work would be required to fully assess any direct correlation between ACJE and social deprivation. Targeting these risks as possible means to predict and reduce the incidence and prevalence of problematic shoulder conditions, including ACJ OA, would be of benefit to public health.

Surgical options for symptomatic ACJ abnormalities are the open distal clavicle excision, or arthroscopic (superior or bursal approach) distal clavicle excision. Excision of the distal clavicle for degenerative change within the ACJ has been shown to be effective, with an 80% to 100% excellent or good outcome by either open or arthroscopic resection.^20^ Several studies have found similar long-term outcomes following either open or arthroscopic ACJE, but faster return to activities in the arthroscopic groups.^21,22^ We discovered that patients undergoing open ACJE were younger than patients undergoing arthroscopic ACJE in our population demographic. Possible reasons for this may pivot on the different in pathology. Open ACJE is considered a quicker and less technically demanding procedure. Perhaps in patients where there is no need to perform adjunctive diagnostic or therapeutic arthroscopy, an open procedure may suffice. Furthermore, the incidence of traumatic and sport-related ACJ disease are more common in younger patients. The incidence of common pathologies such as rotator cuff tears and subacromial impingement syndrome is similar to that of the arthroscopic ACJE age demographic. This may lead to a higher proportion of younger symptomatic patients easier to clinically classify an as isolated ACJ OA or osteolysis, subsequently being offered an open ACJE.

There are several limitations to this study. Variance in individual surgeon practice may have impacted the data. Also, these data only represent findings in one geographical region. The population data did not account for annual changes in population. Additionally, we acknowledge that surgery to excise the AC joint in isolation may be quite different to the group of patients that need shoulder acromioplasty (open or arthroscopic) in addition to AC joint resection in the same setting and further work would be required to investigate isolated arthroscopic ACJE versus isolated open ACJE to draw any definitive conclusion between these groups . The data relied on accurate coding and the retrospective nature of this study leaves itself open to this type of error. The study attempted to quantify changes in practice – this is difficult to prove given multiple parameters that may have influenced change.

## Conclusion

Symptomatic ACJ OA is a common problem for which there is an increasing incidence of surgical treatment, specifically arthroscopic ACJE. This increase is paralleled by a similar increase in arthroscopic RCR and SAD. Future studies to determine specific reasons may affect public and occupational health planning.
